# Functional Metagenomics: A High Throughput Screening Method to Decipher Microbiota-Driven NF-κB Modulation in the Human Gut

**DOI:** 10.1371/journal.pone.0013092

**Published:** 2010-09-30

**Authors:** Omar Lakhdari, Antonietta Cultrone, Julien Tap, Karine Gloux, Françoise Bernard, S. Dusko Ehrlich, Fabrice Lefèvre, Joël Doré, Hervé M. Blottière

**Affiliations:** 1 INRA, UMR 1319 Micalis, Jouy-en-Josas, France; 2 LibraGen S.A., Toulouse, France; Louisiana State University, United States of America

## Abstract

**Background/Aim:**

The human intestinal microbiota plays an important role in modulation of mucosal immune responses. To study interactions between intestinal epithelial cells (IECs) and commensal bacteria, a functional metagenomic approach was developed. One interest of metagenomics is to provide access to genomes of uncultured microbes. We aimed at identifying bacterial genes involved in regulation of NF-κB signaling in IECs. A high throughput cell-based screening assay allowing rapid detection of NF-κB modulation in IECs was established using the reporter-gene strategy to screen metagenomic libraries issued from the human intestinal microbiota.

**Methods:**

A plasmid containing the secreted alkaline phosphatase (SEAP) gene under the control of NF-κB binding elements was stably transfected in HT-29 cells. The reporter clone HT-29/kb-seap-25 was selected and characterized. Then, a first screening of a metagenomic library from Crohn's disease patients was performed to identify NF-κB modulating clones. Furthermore, genes potentially involved in the effect of one stimulatory metagenomic clone were determined by sequence analysis associated to mutagenesis by transposition.

**Results:**

The two proinflammatory cytokines, TNF-α and IL-1β, were able to activate the reporter system, translating the activation of the NF-κB signaling pathway and NF-κB inhibitors, BAY 11-7082, caffeic acid phenethyl ester and MG132 were efficient. A screening of 2640 metagenomic clones led to the identification of 171 modulating clones. Among them, one stimulatory metagenomic clone, 52B7, was further characterized. Sequence analysis revealed that its metagenomic DNA insert might belong to a new *Bacteroides* strain and we identified 2 loci encoding an ABC transport system and a putative lipoprotein potentially involved in 52B7 effect on NF-κB.

**Conclusions:**

We have established a robust high throughput screening assay for metagenomic libraries derived from the human intestinal microbiota to study bacteria-driven NF-κB regulation. This opens a strategic path toward the identification of bacterial strains and molecular patterns presenting a potential therapeutic interest.

## Introduction

The intestinal tract harbors trillions of commensal bacteria representing over a thousand species and encoding over one hundred and fifty fold more genes than the human genome. During the past decade, the gut microbiota was revealed as providing an important functional contribution to its host physiology and in maintenance of health [1]. However, the understanding of these functional interactions is in its infancy. The limitation imposed by the inability to cultivate the majority of the indigenous microbial species is now bypassed with the metagenomic approach [2]. Metagenomic libraries already allowed sequence-based explorations of the human intestinal microbiome that drew up microbial genomic and genetic diversity [3], [4], [5]. Furthermore, the metagenomic approach has been used for some functional investigations of gut microbial communities [6], [7], [8], [9], however, these applications are few compared to metagenomic studies of other environmental ecosystems [for review: 2], [10].

The human intestinal microbiota has been shown to participate in epithelium maturation, host nutrition and protection against pathogens [11] and more recently in regulating gut epithelial cells proliferation [12], host energy metabolism [13] and immune responses [14], thus reflecting the symbiotic and beneficial crosstalk between intestinal epithelial cells (IECs) and intestinal microbiota. However, there is also evidence implicating microbiota in diseases such as allergies [15], inflammatory bowel disease (IBD) [16] and cancer [17]. This evidence unmasks the unwanted face of the microbiota and stresses the importance of an accurate equilibrium between microbes and mucosal immune system. Indeed, the mucosa has the challenge to sense pathogens while being unresponsive to food antigens and commensals, in order to maintain integrity and normal function of the intestine. Traditionally, immune responses in the gut involved the gut-associated lymphoid tissue (GALT), but it is now established that IECs are also an essential component in innate immunity [18]. Theses cells can directly sense commensal bacteria and pathogens *via* pattern recognition receptors (PRRs), including Toll-like receptors (TLRs) and Nod-like receptors (NLRs). These receptors are specialized in the recognition of conserved bacterial and viral structures and generally activate proinflammatory pathways warning the host about infections [19].

Immune and inflammatory responses in the gut involve the transcription factor NF-κB. This DNA binding protein is the transcriptional factor of an evolutionarily conserved regulatory pathway that drives expression of a large number of genes involved in proinflammatory processes at the site of infection or tissue damage. It also controls cell survival, proliferation and differentiation gene expression induced by a wide range of noxious stimuli [20], [21]. Recent studies have demonstrated that NF-κB signaling is a critical element of the homeostatic immuno-inflammatory function in the gut [22] and both deficiency in, or hyperactivation of this transcription factor are linked with pathologies such as chronic IBD or obesity [23], [24], [25], [26], [27].

Thus, determining the factors that regulate this key pathway is of great scientific and clinical interest. In the present study, we present the development of a high throughput functional screening method of metagenomic libraries designed to explore novel NF-κB modulatory potentials within the human intestinal microbiota. A cellular tool, based on the reporter gene strategy, was obtained using the intestinal epithelial cell line HT-29, and was validated and applied for screening of a metagenomic library issued from the intestinal microbiota of Crohn's disease (CD) patients [4]. This strategy opens a way toward identification of bacterial species and effectors involved in the interaction with IECs *via* NF-κB signaling.

## Materials and Methods

### Cell culture

To construct the reporter model, we selected the human colorectal carcinoma cell line HT-29 that was obtained from the American Type Culture Collection (Rockville, MD). HT-29 cells were grown in RPMI 1640 medium (Sigma) with 2 mM L-glutamine, 50 IU/mL penicillin, 50 µg/mL streptomycin and 10% heat-inactivated fetal calf serum (FCS - Lonza) in a humidified 5% CO_2_ atmosphere at 37°C. The THP-1 blue™ CD14+ cells used as control for TLRs responses were obtained from Invivogen and used according to the manufacturer's instruction.

### Construction of stable NF-κB reporting HT-29 cells

Stable HT-29 transfectants containing the secreted alkaline phosphatase (SEAP) reporter gene were obtained after cell transfection with the reporter plasmid pNiFty2-SEAP (Invivogen), which contains SEAP as reporter gene and zeocin as resistance antibiotic gene, using TFX-50™ (Promega) following manufacturer's instruction. Cells were cultured for 3 weeks under zeocin (50 µg/mL) selection and cloned. The HT-29/kb-seap-25 clone was selected for its response to 10 ng/ml of TNF-α after 24 h stimulation.

### Analyses of NF-κB activation

For each experiment, HT-29/kb-seap-25 reporter cells were seeded at 50 000 cells per well, into 96-wells plates and incubated 24 hours before stimulation. Cells were stimulated with 10 µl of each tested substances for a final volume per well of 100 µl.

SEAP in the supernatant was revealed using Quanti-Blue™ reagent (Invivogen) according to the manufacturer's protocol and quantified as OD at 655 nm. All measurements were performed using a microplate reader (Infinite 200, Tecan).

### NF-κB inhibitors

NF-κB inhibitors caffeic acid phenethyl ester (CAPE), BAY 11-7082 ((E)3-[(4-methylphenyl)sulfonyl]-2-propenenitrile) and MG132 (ZLeu-Leu-Leu-H) were provided by Calbiochem. Cells were stimulated with a concentration range of each inhibitor in the presence or absence of 10 ng/mL of TNF-α.

### TLRs analysis

#### TLRs ligands response profiles

The TLR response profile was determined using the TLR1-9 agonist kit (Invivogen) according to manufacturer's instruction. Ligands and working concentrations are for TLR1/2: Pam3CSK4 (1 µg/mL); TLR2: Heat Kiled *Listeria monocytogenes* (10^8^ cells /mL); TLR3: Poly(I:C) (10 µg/mL); TLR4: *E. coli K12* LPS (10 µg/mL); TLR5: *Salmonella typhimurium* Flagellin (10 µg/mL); TLR6/2: FSL1 (1 µg/mL); TLR7: Imiquimod (1 µg/mL); TLR8: ssRNA40 (1 µg/mL); TLR9: ODN2006 (5 µM). **TLRs expression profiles by flow cytometry.** Reporter cells were washed with 2% FCS in phosphate-buffered saline (PBS) and stained with TLRs antibodies (Ab) for 30 min at 4°C. Ab for human TLR2, TLR3, TLR4, and TLR9 were obtained from eBioscience. TLR5, TLR6 and TLR7 Ab were from Imgenex and TLR8 antibody from Axxora. Isotype matched Ab were used as negative controls and all antibodies used were originally coupled with Phycoerythrin (PE). After incubation, cells were washed again with PBS+2% FCS and fixed (CellFix – BD bioscience) prior to analysis using a Becton-Dickinson FACSCalibur flow cytometer and CellQuest software (BD Biosciences). For evaluation of total (surface plus intracellular) expression, cells were fixed and permeabilized using CytoFix/CytoPerm (BD Pharmingen). For each measurement, 3×10^4^ cells were analysed.

### High throughput screening of metagenomic clones

We used the HT-29/kb-seap-25 cells to screen a metagenomic library issued from the intestinal microbiota of CD patients [4] for NF-κB modulatory capacities. The screened library consisted of 2640 *Escherichia coli* (*E. coli*) DH10B clones bearing fosmids with metagenomic inserts of 40 kb length approximately randomly selected from a global library of 25 000 clones. *E. coli* bearing a fosmid without a metagenomic insert was used as control (referred as metagenomic controls). Metagenomic clones were cultured for 24 hours in lysogeny broth (LB) medium in 96 wells plates. Then OD_600 nm_ was measured and given as a raw value. It is important to precise that this OD value corresponds to a measurement in 96 well plates and a culture volume of 150 µl. In these conditions, growth stationary phase of *E. coli* started at 0.4 OD approximately. Bacterial cells were broken with glass beads (106 µm, Sigma) in a mixer mill (15 oscillations/second, two cycles of 90 sec). The suspension was filtered by centrifugation at 4°C through a 0.2 µm microplate filter (Corning) and added to HT-29/kb-seap-25 reporter clone at 10% vol/vol to a final volume of 100 µl. Seeding of the reporter cells, addition of lysates and SEAP activity measurement were performed using a robotic pipetting workstation (Microlab Star, Hamilton). Ten selected metagenomic clones, 5 stimulatory and 5 inhibitory, were grown further in 8 independent cultures and tested to validate their effect.

### Characterization of the stimulatory clone 52B7

A preparation of supernatant and lysed pellet was done to determine the active culture fraction of 52B7. Supernatant of an overnight culture grown at 37°C was collected after filtration on a 0.2 µm filter. After centrifugation of the bacterial culture at 5000 g/10 min, pellet was resuspended in RPMI and bacterial cells were broken by shaking with glass beads in a mixer mill as described above.

The 52B7 fosmid was purified using the NucleoBond extraction kit (Macherey-Nagel) and the DNA insert was sequenced (Genoscope, Evry, France). The sequence is available on GenBank (accession number HQ231916). Potential transcription units were detected using SoftBerry's software FGENESB (http://linux1.softberry.com/berry.phtml). Potential ORFs contained in the 40 kb insert were determined using GeneMark.hmm for prokaryotes (http://exon.biology.gatech.edu/gmhmm2_prok.cgi) and FGENESB. Both analyses were performed using *Bacteroides thetaiotaomicron* VPI-5482 as model organism. The output predicted protein sequences were further blasted in order to have phylogenetic and function estimation. The final phylogenetic assignment was expressed in percentage of total genes length coverage. The original host was searched using Blastn and the microbial genome database on the NCBI.

To determine the potential gene(s) involved in the stimulatory effect of 52B7, fosmid transposition was performed as previously described [6] using the EZ-Tn5™ <KAN-2> kit according to manufacturer instructions (Epicentre). Two hundred transposed clones of 52B7 were picked and tested for a revertant phenotype toward NF-κB activation. Three transposed clones with a revertant behaviour were validated and transposon insertion site in the metagenomic insert was determined by sequencing (Beckman Coulter Genomics) using primers homologous to the ends of the transposon (Epicentre).

### Statistical analysis

Presented results were representative of a minimum of 3 independent experiments. Results were expressed as mean ± standard deviation of triplicate measurements. Data were analyzed by Student's *t* test.

## Results

### Development and characterization of the NF-κB reporter system in HT-29 cells

**Figure 1:**
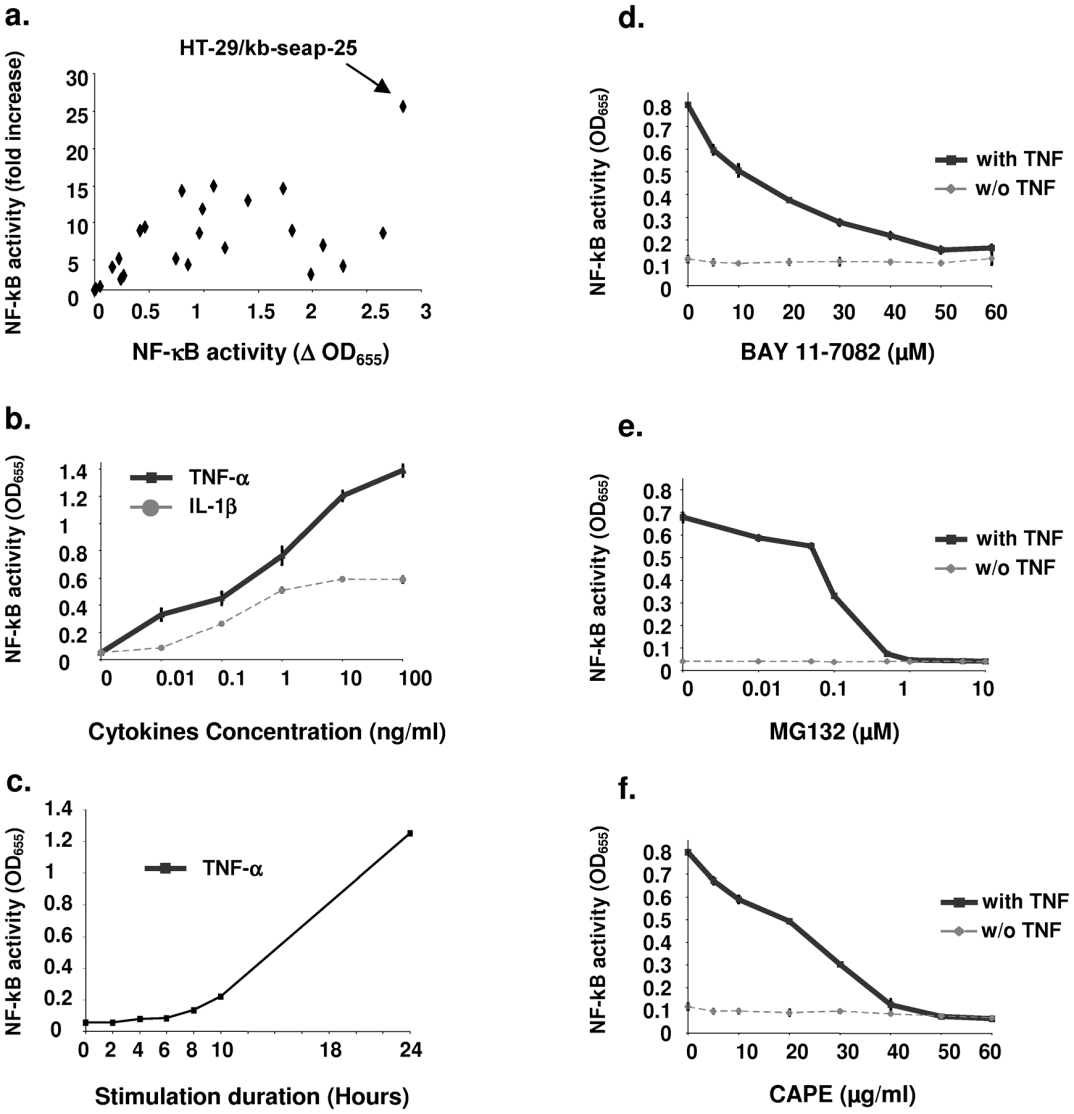
Selection and characterization of a NF-κB reporter clone of HT-29. a. Response of reporter clones to TNF-α stimulation (10 ng/mL). The signal level (OD_655 nm_) and the ratio of signal with and without stimulation are displayed. A highly efficient clone HT-29/kb-seap-25 is highlighted (arrow). b. Dose-response of HT-29/kb-seap-25 cells to increasing concentrations of TNF-α or IL-1β. Reporter gene activity was measured after 24 hours stimulation. c. Kinetic of HT-29/kb-seap-25 cells response to incubation with TNF-α (10 ng/mL) for different times. d–f. Inhibition of the TNF-α induction of the NF-κB reporter gene in the HT-29/kb-seap-25 cells. Three inhibitor, BAY 11-7082 (d.), MG132 (e.) and CAPE (f.) were tested in presence (solid line) or absence (doted line) of TNF-α (10 ng/mL). Cells were analyzed for NF-κB activity after 24 hours. In all cases, SEAP activity was measured in the supernatant using Quanti-Blue™ (Invivogen) and was expressed as OD at 655 nm.

After transfection and culture of several clones under zeocin selection, we selected the best responding reporter clone, HT-29/kb-seap-25, which displayed a high signal upon TNF-α stimulation and a high ratio between unstimulated and stimulated states (Fig. 1a). To determine whether the reporter gene in the selected reporter clone reflects well the regulation of the NF-κB signaling pathway, we characterized extensively its response to known NF-κB modulating molecules, including proinflammatory cytokines, chemical inhibitors and TLRs agonists.

The two proinflammatory cytokines, TNF-α and IL-1β, were able to induce expression of the reporter genes in a dose dependent manner (Fig. 1b). As expected, the induction was stronger with TNF-α than IL-1β and did not reach saturation even at the highest dose used (100 ng/mL), while IL-1β was saturated at 10 ng/mL (Fig. 1b). We examined the kinetics of activation of our NF-κB reporter systems by incubating the cells with TNF-α (10 ng/mL) at different times (Fig. 1c). Activity of the reporter clone HT-29/kb-seap-25 increased in a time dependent manner, with a maximum effect occurring after 24 hours stimulation. A weak activity was detected after 4 hours and continued to increase throughout the experiment (Fig. 1c).

Three chemical inhibitors acting at different level of the NF-κB pathway were tested. Reporter cells were incubated for 24 hours with different concentrations of CAPE, BAY-117082 and MG132, and co-treated with TNF-α. NF-κB activation was reduced by 50% in cells treated with 14.2 µM of BAY 11-7082 (Fig. 1d) or 22.2 µg /mL of CAPE (Fig. 1f). In the case of MG132, IC50 was 0.2 µM (Fig. 1e). No effect on the reporter system was observed when the cells were treated with the solvent (DMSO) alone (not shown).

### TLRs expression and response to TLRs ligands

**Figure 2:**
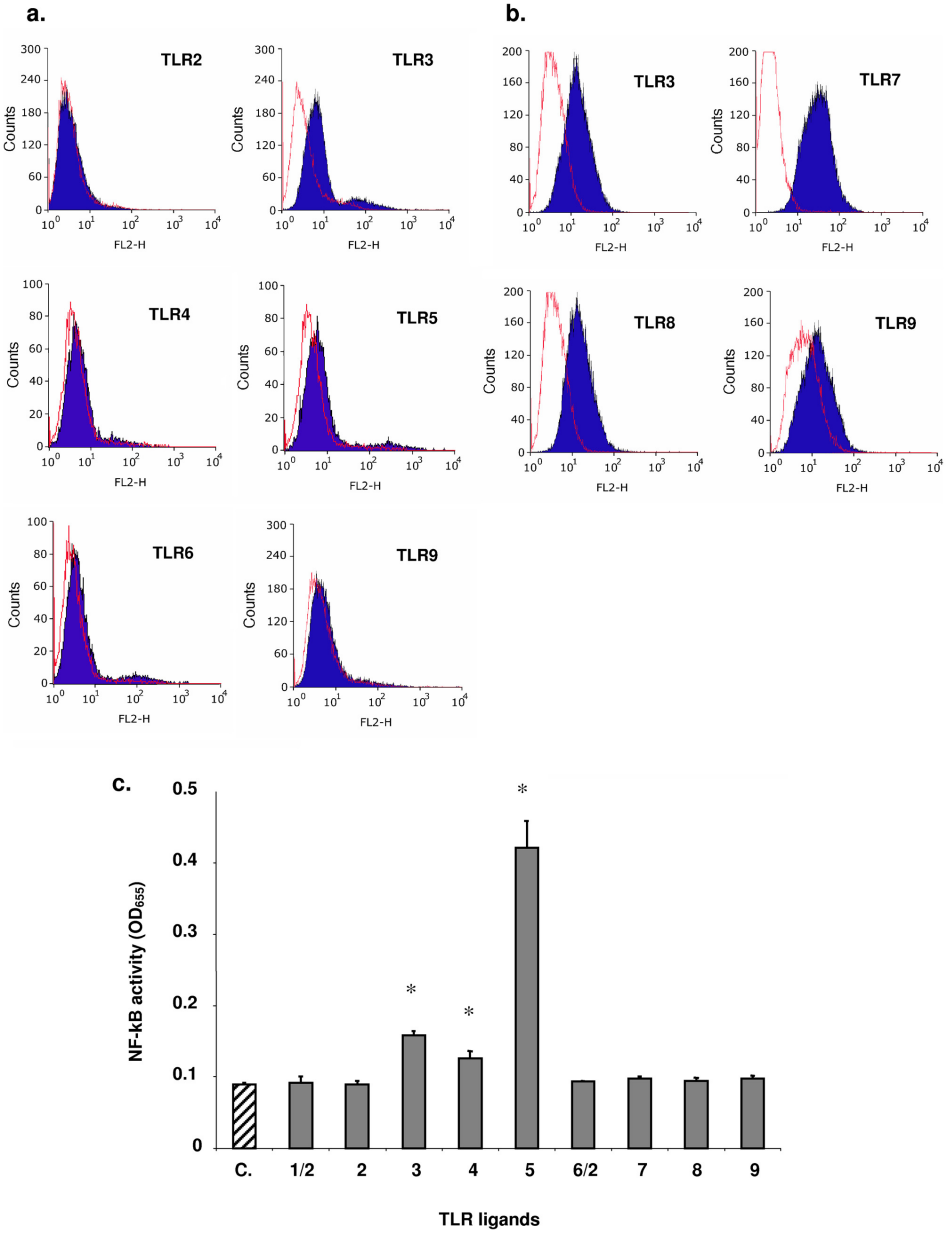
TLRs expression and response to TLRs agonists. Cell surface (a.) and intracellular (b.) expression of different TLRs as assessed by flow cytometry using antibodies to TLR2 to TLR9 (from BD Biosciences) in HT-29/kb-seap-25 cells using a FACScalibur (BD Biosciences). Controls are represented in red. c. Effect of various TLRs ligands on reporter gene activity quantified as OD measured at 655 nm by HT-29/kb-seap-25 cells assessed after 24 hours stimulation. Ligands are for TLR1/2: Pam3CSK4 (1 µg/mL); TLR2: Heat Kiled *Listeria monocytogenes* (10^8^ cells /mL); TLR3: Poly(I:C) (10 µg/mL); TLR4: *E. coli K12* LPS (10 µg/mL); TLR5: *Salmonella typhimurium* Flagellin (10 µg/mL); TLR6/2: FSL1 (1 µg/mL); TLR7: Imiquimod (1 µg/mL); TLR8: ssRNA40 (1 µg/mL); TLR9: ODN2006 (5 µM). All ligands are from Invivogen. *  =  p<0.05.

The TLRs have an immunological sensing role for bacterial, fungal, viral and parasitic organisms. Therefore, we first examined the presence of 8 different TLRs in the HT-29/kb-seap-25 cells (Fig. 2a and 2b). TLR5 was expressed by HT-29 cells however no TLR2 and few TLR4, TLR6 and TLR9 were detected at the cell membrane (MFI given in Table S1). Interestingly, TLR3 expression was detected at cell membrane, but also in the endolysosomal compartment as revealed after permeabilization. As expected, TLR7, TLR8 and TLR9 were only found in the intracellular compartment.

When we tested induction of the reporter gene by 9 different TLRs ligands, we found that TLR3, TLR4 and TLR5 ligands were active, concordantly with the presence of the cognate receptors (Fig. 2b). *S. typhimurium* flagellin was highly stimulatory, therefore correlating with TLR5 expression in these cells, while *E. coli* K12 lipopolysaccharide (LPS) and Poly-IC, ligands for TLR4 and TLR3, respectively, were also stimulatory but to a lesser degree. Despite the fact that TLR7, 8 and 9 were detected by flow cytometry in the cytoplasm of HT-29/kb-seap-25 cells, no SEAP activity was detected when the cells were stimulated with their respective agonist, imiquimod, ssRNA and CpG-ODN.

Finally, the agonists were tested for control on THP-1 blue™ CD14+ which is an NF-κB reporter monocytic cell line (Invivogen). We observed that THP-1 responded to the stimulation with all tested agonists except those which receptors (TLR3, 7, 8 and 9) are located in the intracellular compartment (data not shown).

### Seeking for modulatory metagenomic clones

**Figure 3:**
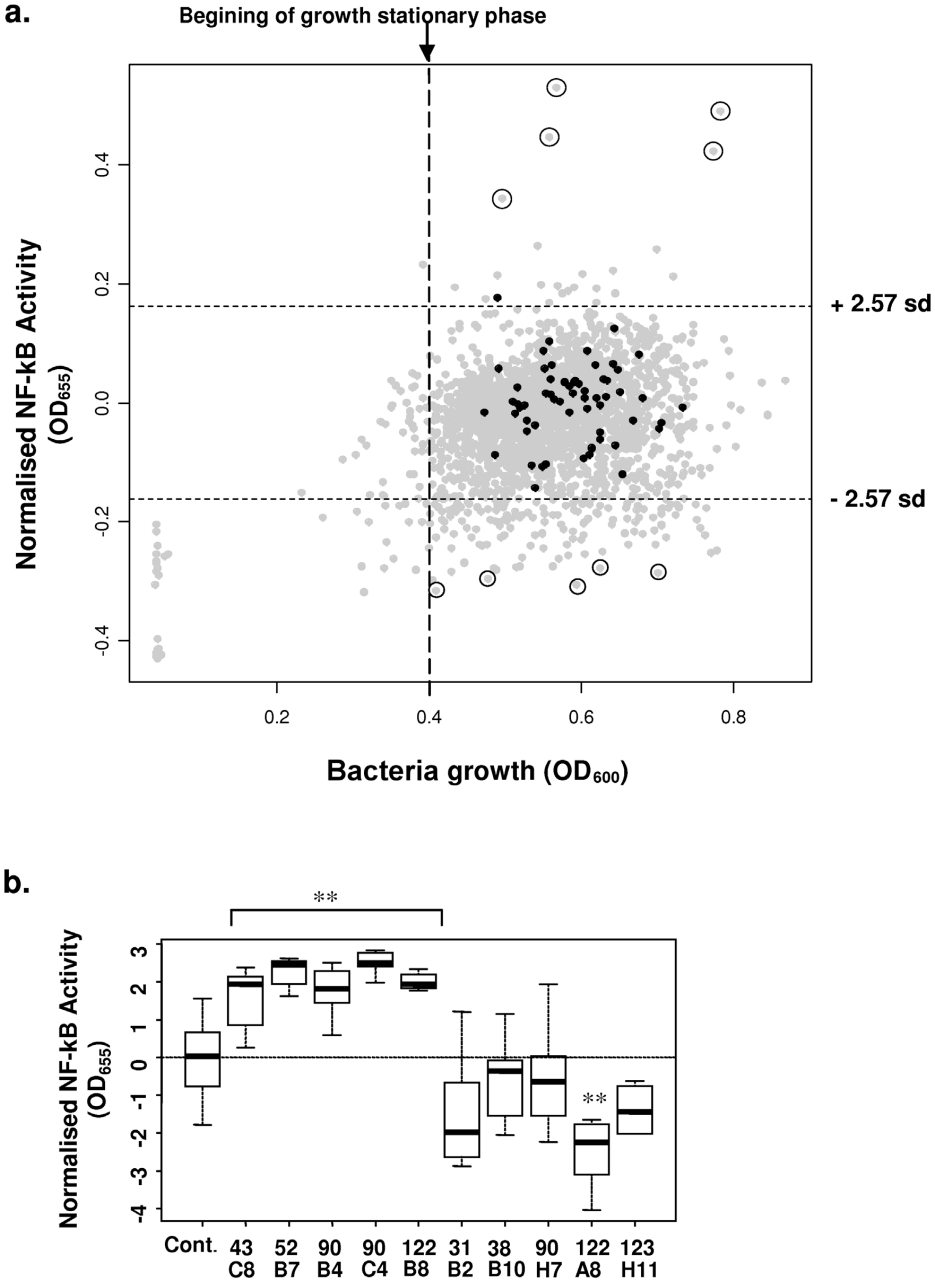
Screening of a metagenomic library for NF-κB modulation using HT-29 reporter cells. a. First screening of 2640 clones of a metagenomic library prepared from the fecal microbiota of CD patients. Lysates were added at 10% vol/vol to HT-29/kb-seap-25. NF-κB activity (expressed as OD_655 nm_) was normalized to the mean activity of the metagenomic controls (*E. coli* DH10B + empty fosmid; represented in black). Bacteria growth was expressed as OD_600 nm_. Standard deviation of the control is represented as horizontal doted line and circle dots represent the 10 active lysates selected for further validation. b. Validation of the effect of 5 activating and 5 inhibiting clones. Metagenomic clones were grown in 8 independent cultures and tested on HT-29/kb-seap-25. Reporter gene activity was assayed after 24 hours stimulation. NF-κB activity (expressed as OD_655 nm_) was normalized to that of the control (*E. coli* DH10B + empty fosmid). ** p<0.01 as compared to control.

NF-κB reporter cells were constructed in view of performing high throughput screening of NF-κB modulation capabilities within metagenomic libraries issued from human intestinal microbiota. A first screening using the HT-29/kb-seap-25 reporter clone was done on 2640 metagenomic clones from a library prepared with the fecal microbiota of CD patients [4]. Results (Fig. 3a) are represented as a plot of reporter gene activity (normalized to that of the metagenomic control) and growth level for each metagenomic clone. In a OD range between 0.4 and 0.8, growth of the metagenomic control was not correlated with NF-κB activity.

A vast majority (93.5%) of the 2640 tested clones had no significant different effect on NF-κB activity as compared to the controls. Interestingly, 22 lysates (0.8%) enhanced the reporter system activity while 149 lysates (5.6%) down-activated it. We decided to validate 10 clones producing the highest enhancing or inhibitory effect (circled on Fig. 3a). These 10 clones were cultivated as 8 independent cultures and tested in a similar way on the HT-29/kb-seap-25 cells (Fig. 3b). All of the 5 activating clones (43C8; 52B7; 90B4; 90C4 and 122B8) displayed high stimulatory effects that were significantly different from the control (p<0.01).

No inhibitory clones affected the expression of the reporter gene to an extent comparable to that of the 5 most stimulatory clones (Fig. 3a). The 5 inhibitory clones selected (31B2; 38B10; 90H7; 122A8 and 123H11) were nevertheless submitted to the validation step and we found one having a very significant and reproducible effect (122A8, Fig. 3b).

This demonstrates that our screening system can successfully identify bacterial metagenomic clones able to modulate NF-κB signaling in HT-29. We also obtained a Caco-2 reporter clone (Caco-2/kb-seap-7) that was characterized similarly to HT-29, but was not used for the present screening (see Method S1, Fig. S1 and Table S5). However, it is noteworthy that on Caco-2 reporter cells, the 10 selected lysates were without effects suggesting potential cell line specificity (data not shown).

### Toward the identification of a new bacterial stimulatory mechanism of NF-κB

For further investigation, we decided to focus on the stimulatory clone 52B7. Metagenomic DNA insert of 52B7 (37,006 base pairs long) contains 43 genes and 23 transcriptional units according to GeneMark.hmm and FGENESB predictions (Table S2). The total predicted genes length represents 80.8% of the total insert length (Table S4). No relatives were found in NCBI bacterial genome database however, the best blastp results of predicted genes for 52B7 insert showed that it is probably issued from a *Bacteroides* genus member (98.5% of predicted genes coverage), the most closely related cultivated strain being *B. vulgatus* ATCC 8482 with 42% of predicted genes coverage (Table S3 and Table S4).

**Figure 4:**
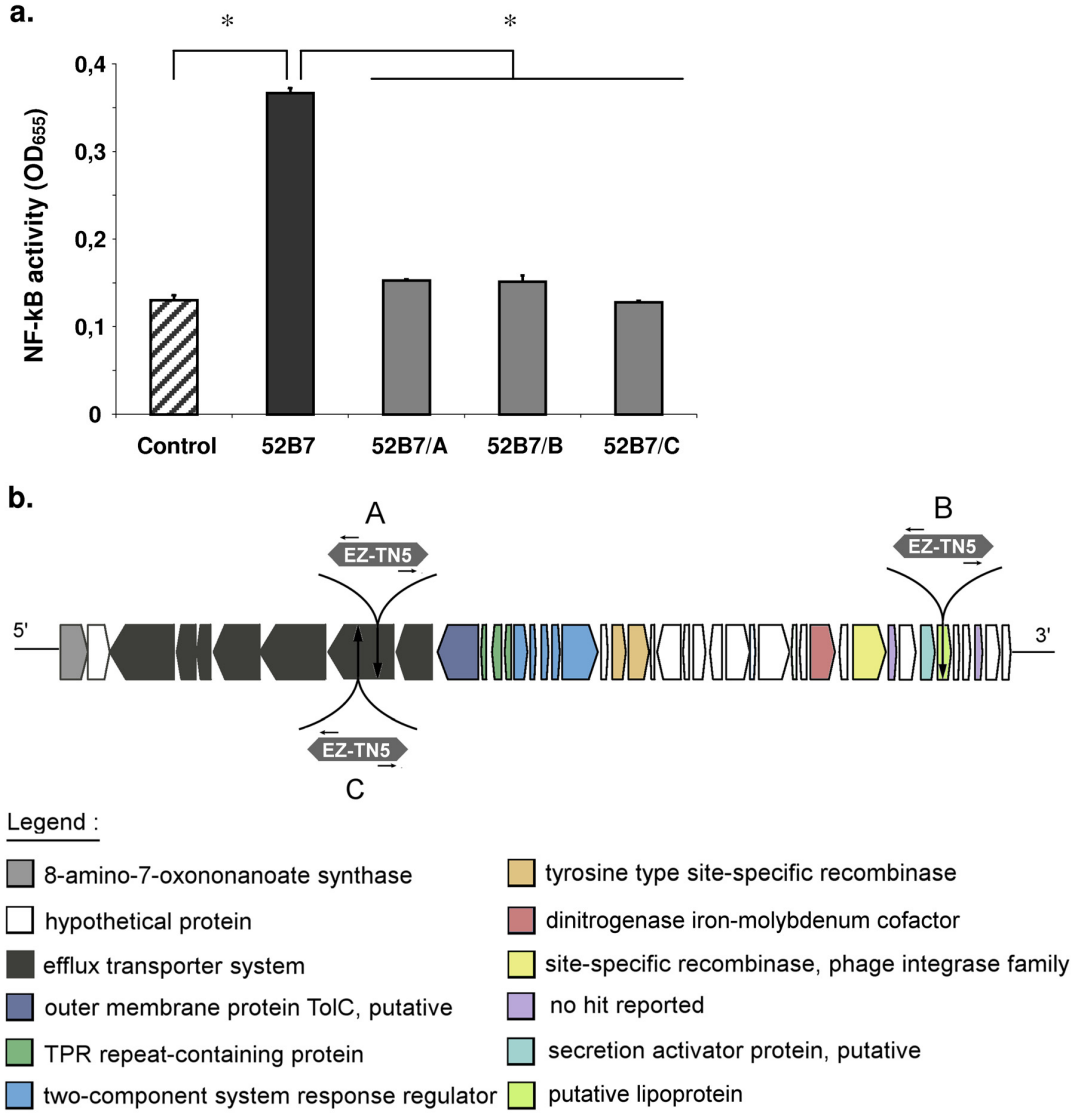
Identification of the bacterial genes involved in the stimulatory effect of 52B7. a. Effect of 3 selected transposed clones with revertant behavior. Filtered culture supernatants were added at 10% vol/vol to HT-29/kb-seap-25. Control corresponds to *E. coli* DH10B bearing empty fosmid. *  =  p<0.05. b. Sequence annotation and localization of transposon insertion sites. EZ-TN5 transposon is represented as a grey box. A, B and C corresponds to the insertion region of the transposed clones 52B7/A, 52B7/B and 52B7/C, respectively.

We then identified the most active fraction by testing separately supernatant and lysed pellet. We observed that the stimulatory effect was mainly present in the supernatant while only a weak activity was detected in the lysed pellet (data not shown). Moreover, we also confirmed that 52B7 supernatant significatively induced IL-8 secretion in both parental HT-29 and HT-29/kb-seap-25 reporter cells as compared with the metagenomic control (Method S2 and Fig. S2). Therefore, we managed to identify the gene(s) or loci responsible for the stimulatory effect and performed mutagenesis on the fosmid by insertion of transposon EZ-TN5. Out of 192 mutants, we obtained 3 transposed clones from 52B7 of which the supernatant did not produce any more stimulation of NF-κB (Fig. 4a). Insertion regions of the transposons were determined by sequencing. Analysis of both insert annotation and sequences from the EZ-TN5 transposon revealed that in 52B7/A and 52B7/C, the transposons were inserted in the same gene encoding an ATP-binding cassette (ABC) transporter permease (Fig. 4b and 8^th^ gene in Table S2). In 52B7/B transposon targeted the 38^th^ gene, which encodes for a putative lipoprotein with unknown function (Table S2). The two targeted genes were part of two putative distinct transcription units (Table S2). Each of the two targeted insert loci was therefore involved in 52B7 stimulatory effect of NF-κB in HT-29.

## Discussion

We have presented the construction and validation of a cell-based screening system to study NF-κB modulation in IECs and its first utilization within screening of metagenomic libraries from the human gut microbiota. Using the reporter gene strategy, we have obtained an HT-29 reporter cell clone (HT-29/kb-seap-25) that allows rapid examination of NF-κB activity regulation. A validation of the selected reporter clone was performed to check the robustness of its response to known activators or inhibitors of NF-κB pathway. We observed a dose-dependent stimulation of NF-κB in HT-29 clone upon treatment with the proinflammatory cytokines TNF-α and IL-1β. These observations are classical and well described in the literature [28]. We tested 3 different NF-κB inhibitors, each one acting at different levels of the NF-κB pathway. Previous studies demonstrated that the proteasome inhibitor MG132 blocks TNF-α-induced degradation of IκBα and leads to the accumulation of phosphorylated IκBα [29]. Since it inhibits proteasome activity, MG132 is not a specific NF-κB inhibitor but is widely used as such. CAPE is an active molecule purified from honeybee hives and was also described as an anti-inflammatory molecule acting at the level of DNA binding in human histiocytic cell line [30]. BAY 11-7082 is an irreversible inhibitor of IκBα phosphorylation thereby sequestering NF-κB in an inactive state in the cytoplasm and preventing activation of downstream signaling [31]. These three molecules were efficient in the HT-29 reporter cells.

The gut epithelium is exposed to numerous microbes without developing uncontrolled inflammation. One of the proposed mechanisms explaining such epithelium tolerance towards commensals is its hypo-responsiveness to bacterial patterns involving TLRs signaling cascade. For example, Toll inhibitory protein (Tollip) was shown to inhibit IL-1R activated kinase (IRAK) activation, resulting in loss of TLR2 and TLR4 response in the gut [32], [33]. In addition, the luminal surface seems to be devoid of TLR2 and 4, their expression being confined to crypt cells [34]. It is noteworthy that characterization of TLRs showed unresponsiveness of our HT-29 and Caco-2 reporter cells toward agonists of the bacterial cell wall components receptors TLR1/2, TLR2 and TLR6/2. Using mRNA and protein analysis, other laboratories [35], [36] showed that both cell lines expressed very low levels of TLR2 mRNA as compared to a TLR2-ligand responsive monocyte cells THP-1. TLR1 and 6 mRNA were not expressed by Caco-2 whereas HT-29 did. In addition, it was demonstrated that these cell lines expressed increased levels of Tollip, the TLR2 signaling inhibitor, which could explain the lack of response obtained with TLR1/2, TLR2 and TLR 6/2 agonists. Challenging HT-29 cells with LPS alone resulted in a small but significant induction of NF-κB-dependent reporter gene activity which is consistent with previous results demonstrating that parental HT-29 line expresses small levels of TLR4 mRNA and protein [35], [37], and high level of Tollip [38]. By contrast, we found that HT-29 reporter cells were strongly responsive to flagellin treatment. TLR5 recognition of bacterial flagellin from both Gram-positive and Gram-negative bacteria was previously reported [39] and was described to be constitutively expressed at the apical and basolateral side in HT-29 [40].

Nucleic acid recognition is possible *via* TLR3, TLR7, TLR8 and TLR9, which are classically located in the intracellular endosomal compartment. Following treatment with Poly-I:C, a synthetic analogue of double stranded RNA, which is recognized by TLR3 [41], NF-κB activation was detected in HT-29 cells but not in THP-1 control reporter cells. This difference could be linked to the localization of TLR3 in HT-29. Indeed expression patterns described for this cell line suggest localization in both cytoplasm and cell membrane [34], [42], [43], a finding that we confirmed by flow cytometry. In our hands, TLR7, TLR8 and TLR9 agonists did not induce NF-κB activation in HT-29, Caco-2 nor in THP1 reporter cell lines.

Validation of our reporter screening tools showed reproducible responses consistent with the literature and the reporter gene strategy proposed is robust for high throughput identification of bacterial modulatory potentials within metagenomic libraries. Metagenomic is a recent approach designed to uncover novel genes and related functions [2]. However, this method can identify only a small proportion of genes from the natural pool due to cloning and heterologous expression limitations in *E. coli*. Nevertheless, *E. coli* is a reliable host since it was shown to be able to express up to 40% of the functional potential from randomly cloned environmental DNA [44]. To fulfill these limitations, work is in progress to generate libraries in a Gram + recipient host. Nevertheless, the methodology presented allowed us to identify 171 metagenomic clones able to modulate NF-κB pathway. Here we presented the first results obtained on the characterization of the stimulatory metagenomic clone 52B7.

The 52B7 insert is issued from a probably unknown *Bacteroides* strain and encodes number of hypothetical proteins with unknown functions. This demonstrates the importance of the functional metagenomic approach in a better understanding of the interactions between a large portion of uncultivated bacteria and their host. Interestingly, the closest relative species was *B. vulgatus*, a commensal bacteria previously found to be more abundant in CD patients than in healthy subjects [45]. Moreover, presence of antibodies against *B. vulgatus* was reported in CD patients [46]. However no mechanism was yet proposed to explain a possible implication of *B. vulgatus* in CD. Concerning the 52B7 insert, no proteins already known to activate NF-κB signaling (such as a TLR ligand) were detected, suggesting the involvement of potentially new stimulatory effectors.

Using mutagenesis by transposition, we identified two different candidate loci responsible for the stimulatory effect of NF-κB on HT-29. The first locus, related to an efflux ABC-type transport system, includes a permease particularly targeted by the transposition experiments and the second locus highlighted includes a putative lipoprotein with unknown function. The contribution of the 2 loci on the stimulatory effect still needs to be elucidated. According to the literature, some ABC transporter proteins ensuring lipoprotein trafficking in Gram-negative bacteria were described [47]. In addition, lipoproteins from pathogenic bacteria are known inducer of immune responses during infection, generally through interaction with TLR2 [48], [49]. It is therefore tempting to assume a potential mechanism in which the efflux ABC transporter would drive the release of the putative lipoprotein, the latter being the bacterial effector responsible for activation of NF-κB in HT-29, potentially through a TLR2-independent manner since this receptor is absent. Future investigations will be necessary to precisely determine bacterial and eukaryotic effectors and mechanisms involved as well as their potential implication in the existing relationship between some commensals (as *B. vulgatus*) and pathogenesis of IBD.

Our approach provided other excellent candidate metagenomic clones for investigation of novel host-commensal interaction mechanisms and these clones are currently being characterized using a similar strategy.

In future work, the NF-κB reporter cell lines will be used in the automated screening of new metagenomic libraries in the frame of the MetaHIT project. This approach targeting NF-κB pathway is currently under development for other signaling pathways including AP1, p53, c-Myc and PPARγ, as well as for genes of interest including Thymic Stromal Lymphopoietin (TSLP) and angiopoietin-like protein 4/fasting-induced adipose factor (Angptl4/FIAF). We think that our functional metagenomic strategy will allow identification of genes that human-associated intestinal microbes may use to affect expression of genes and pathways of their host, thus opening new avenues towards a better understanding of microbiota-host interactions and potential development of new probiotics or therapeutic molecules.

## Supporting Information

Figure S1 Characterization of Caco-2/kb-seap-7 cell clone. a. Dose response. Caco-2/kb-seap-7 cells were stimulated with increasing concentration of TNF-α or IL-1β. Reporter gene activity quantified as OD measured at 655 nm was measured after 24 hours stimulation. b. Response to TLRs ligands. Caco-2/kb-seap-7 clone was stimulated with different TLRs agonists as described in Figure 2. NF-κB activity was measured after 24 hours stimulation. c–e. Dose-dependent effect of BAY 11-7082 (c), MG132 (d) and CAPE (e) on NF-κB inhibition was tested in presence (solid line) or absence (doted line) of IL-1β. Culture supernatant were analyzed for NF-κB activity after 24 hours treatment. Results are mean ± standard deviation of triplicate measurements for one representative of three independent experiments.
(0.24 MB TIF)

Figure S2 52B7 supernatant stimulates IL-8 secretion in HT-29 cells. Effect of filtered bacterial supernatant of 52B7 on parental HT-29 (a) and HT-29/kb-seap-25 (b). Supernatant was added at 10% vol/vol and IL-8 secretion was measured after 24 hours incubation. Control corresponds to culture supernatant from the metagenomic control (*E. coli* bearing empty fosmid). Bacteria growth medium corresponds to LB. TNF-α (10 ng/mL) was used as positive control. Results are expressed as mean ± standard deviation. One representative experiment of 2 independent experiments is shown. *  =  p<0.05.
(0.25 MB TIF)

Table S1 TLRs expression in HT-29/kb-seap-25. Results are expressed as MFI (Mean Fluorescence Intensity).
(0.03 MB DOC)

Table S2 Functional annotation of the 52B7 metagenomic insert. * T.U  =  transcription unit. Length is expressed in bp (base pairs).
(0.11 MB DOC)

Table S3 Phylogenetic indication of the first blastp result for predicted genes of 52B7 insert. Length is expressed in bp (base pairs).
(0.06 MB DOC)

Table S4 The 52B7 metagenomic insert, key facts and figures. * bp: base pairs.
(0.03 MB DOC)

Table S5 TLRs expression in Caco-2/kb-seap-7. Results are expressed as MFI (Mean Fluorescence Intensity).
(0.03 MB DOC)

Method S1 Construction and use of Caco-2 reporter cells.
(0.03 MB DOC)

Method S2 Measurment of IL-8 secretion.
(0.02 MB DOC)
