# Correction: Functional Metagenomics: A High Throughput Screening Method to Decipher Microbiota-Driven NF-κB Modulation in the Human Gut

**DOI:** 10.1371/annotation/4ea12169-7c97-497c-a45f-52203543065f

**Published:** 2010-10-12

**Authors:** Omar Lakhdari, Antonietta Cultrone, Julien Tap, Karine Gloux, Françoise Bernard, S. Dusko Ehrlich, Fabrice Lefèvre, Joël Doré, Hervé M. Blottière

In the Results subsection "TLRs expression and response to TLRs ligands," the incorrect figure panel was cited in the first sentence. The sentence should read: "When we tested induction of the reporter gene by 9 different TLRs ligands (Fig. 2c), we found that TLR3, TLR4 and TLR5 ligands were active, concordantly with the presence of the cognate receptors."

