# Correction: Functional Metagenomics: A High Throughput Screening Method to Decipher Microbiota-Driven NF-κB Modulation in the Human Gut

**DOI:** 10.1371/annotation/9f1b7f00-bcc0-4442-9775-491ebdafc7bc

**Published:** 2010-10-12

**Authors:** Omar Lakhdari, Antonietta Cultrone, Julien Tap, Karine Gloux, Françoise Bernard, S. Dusko Ehrlich, Fabrice Lefèvre, Joël Doré, Hervé M. Blottière

In the Materials and Methods subsection "Characterization of the stimulatory clone 52B7," the second sentence of the last paragraph was incorrect. The correct sentence should read: "One hundred and ninety two transposed clones of 52B7 were picked and tested for a revertant phenotype toward NF-κB activation."

