# Supplementary figures and images for: Functional Metagenomics: A High Throughput Screening Method to Decipher Microbiota-Driven NF-κB Modulation in the Human Gut
Source: PLoS One. 2010 Sep 30;5(9):e13092. doi: 10.1371/journal.pone.0013092 (PMC2948039; doi:10.1371/journal.pone.0013092)

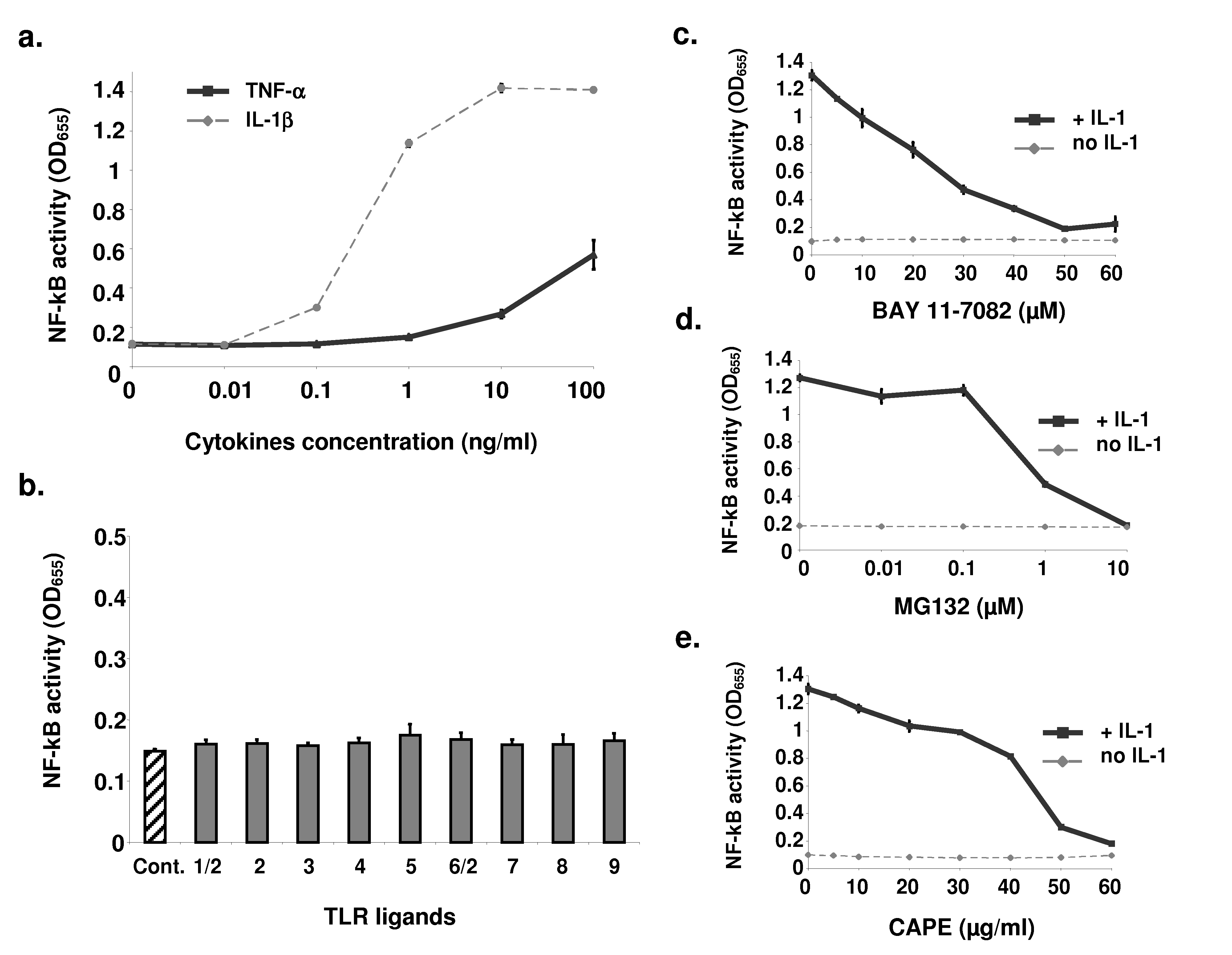

Supplement: Figure S1 — Characterization of Caco-2/kb-seap-7 cell clone. a. Dose response. Caco-2/kb-seap-7 cells were stimulated with increasing concentration of TNF-α or IL-1β. Reporter gene activity quantified as OD measured at 655 nm was measured after 24 hours stimulation. b. Response to TLRs ligands. Caco-2/kb-seap-7 clone was stimulated with different TLRs agonists as described in Figure 2. NF-κB activity was measured after 24 hours stimulation. c–e. Dose-dependent effect of BAY 11-7082 (c), MG132 (d) and CAPE (e) on NF-κB inhibition was tested in presence (solid line) or absence (doted line) of IL-1β. Culture supernatant were analyzed for NF-κB activity after 24 hours treatment. Results are mean ± standard deviation of triplicate measurements for one representative of three independent experiments. (0.24 MB TIF) [file pone.0013092.s001.tif]

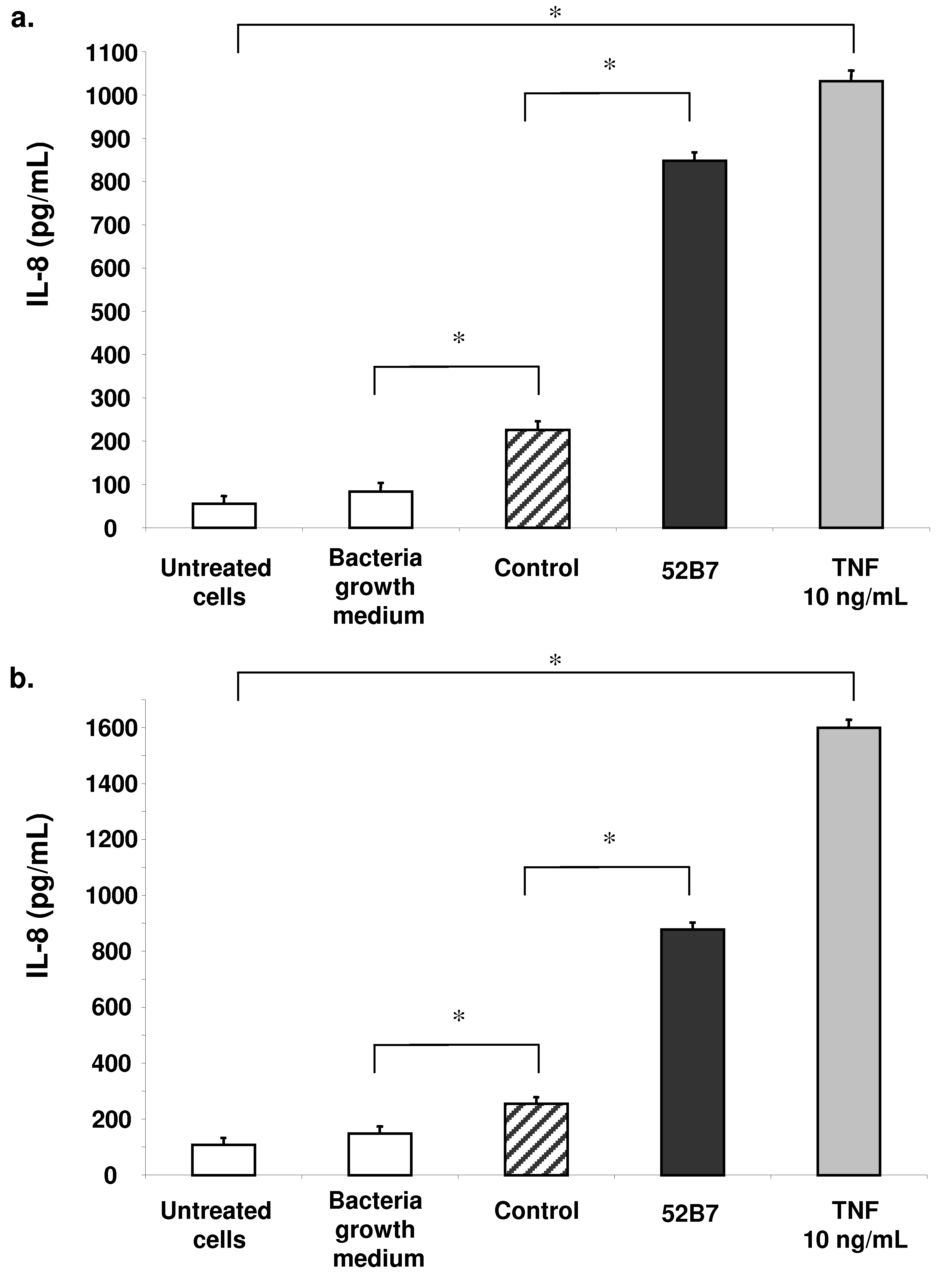

Supplement: Figure S2 — 52B7 supernatant stimulates IL-8 secretion in HT-29 cells. Effect of filtered bacterial supernatant of 52B7 on parental HT-29 (a) and HT-29/kb-seap-25 (b). Supernatant was added at 10% vol/vol and IL-8 secretion was measured after 24 hours incubation. Control corresponds to culture supernatant from the metagenomic control (E. coli bearing empty fosmid). Bacteria growth medium corresponds to LB. TNF-α (10 ng/mL) was used as positive control. Results are expressed as mean ± standard deviation. One representative experiment of 2 independent experiments is shown. * = p<0.05. (0.25 MB TIF) [file pone.0013092.s002.tif]
