# Supplementary material for: Functional Metagenomics: A High Throughput Screening Method to Decipher Microbiota-Driven NF-κB Modulation in the Human Gut
Source: PLoS One. 2010 Sep 30;5(9):e13092. doi: 10.1371/journal.pone.0013092 (PMC2948039; doi:10.1371/journal.pone.0013092)
Supplement: Table S1 — TLRs expression in HT-29/kb-seap-25. Results are expressed as MFI (Mean Fluorescence Intensity). (0.03 MB DOC) [file pone.0013092.s003.doc]

Table S1. TLRs expression in HT-29/kb-seap-25.

| TLR | Membrane | Isotype Control (membrane) | Intracellular | Isotype Control  (intracellular) |
| --- | --- | --- | --- | --- |
| TLR2 | 5.83 | 6.53 |  |  |
| TLR3 | 20.43 | 4.24 | 17.44 | 3.68 |
| TLR4 | 7.66 | 6.53 |  |  |
| TLR5 | 21.51 | 6.53 |  |  |
| TLR6 | 10.54 | 4.24 |  |  |
| TLR7 |  |  | 11.96 | 3.24 |
| TLR8 |  |  | 18.48 | 3.68 |
| TLR9 | 11.66 | 4.56 | 17.60 | 3.55 |
