# Supplementary material for: Functional Metagenomics: A High Throughput Screening Method to Decipher Microbiota-Driven NF-κB Modulation in the Human Gut
Source: PLoS One. 2010 Sep 30;5(9):e13092. doi: 10.1371/journal.pone.0013092 (PMC2948039; doi:10.1371/journal.pone.0013092)
Supplement: Table S2 — Functional annotation of the 52B7 metagenomic insert. * T.U = transcription unit. Length is expressed in bp (base pairs). (0.11 MB DOC) [file pone.0013092.s004.doc]

Table S2. Functional annotation of the 52B7 metagenomic insert.

| **Gene** | **Strand** | **Left End** | **Right End** | **Length** | **T.U*** | **Description** | **E-value** |
| --- | --- | --- | --- | --- | --- | --- | --- |
| 1 | + | 1 | 1032 | 1032 | 1 | >ref|YP_001300420.1| 8-amino-7-oxononanoate synthase [Bacteroides vulgatus ATCC 8482] | 0 |
| 2 | + | 1120 | 2007 | 888 | 1 | >ref|YP_001300421.1| hypothetical protein BVU_3167 [Bacteroides vulgatus ATCC 8482] | 5,0E-163 |
| 3 | - | 2015 | 4366 | 2352 | 2 | >ref|ZP_06742050.1| efflux ABC transporter, permease protein [Bacteroides vulgatus PC510] | 0 |
| 4 | - | 4426 | 5100 | 675 | 2 | >ref|YP_001300423.1| putative ABC transporter ATP-binding protein [Bacteroides vulgatus ATCC 8482] | 2,0E-126 |
| 5 | - | 5120 | 5605 | 486 | 2 | >ref|ZP_06742048.1| efflux ABC transporter, permease protein [Bacteroides vulgatus PC510] | 1E-83 |
| 6 | - | 5602 | 7518 | 1917 | 2 | >ref|ZP_06742048.1| efflux ABC transporter, permease protein [Bacteroides vulgatus PC510] | 0 |
| 7 | - | 7539 | 9908 | 2370 | 2 | >ref|ZP_06742047.1| efflux ABC transporter, permease protein [Bacteroides vulgatus PC510] | 0 |
| 8 | - | 9922 | 12318 | 2397 | 2 | >ref|ZP_06742046.1| efflux ABC transporter, permease protein [Bacteroides vulgatus PC510] | 0 |
| 9 | - | 12329 | 13579 | 1251 | 2 | >ref|ZP_06742045.1| efflux transporter, RND family, MFP subunit [Bacteroides vulgatus PC510] | 0 |
| 10 | - | 13664 | 15133 | 1470 | 2 | >ref|YP_001300428.1| outer membrane protein TolC, putative [Bacteroides vulgatus ATCC 8482] | 0 |
| 11 | - | 15271 | 15441 | 171 | 3 | >ref|YP_001300429.1| TPR repeat-containing protein [Bacteroides vulgatus ATCC 8482] | 7,0E-13 |
| 12 | - | 15495 | 15767 | 273 | 3 | >ref|YP_001300429.1| TPR repeat-containing protein [Bacteroides vulgatus ATCC 8482] | 3,0E-45 |
| 13 | - | 15857 | 16096 | 240 | 3 | >ref|YP_001300429.1| TPR repeat-containing protein [Bacteroides vulgatus ATCC 8482] | 8,0E-39 |
| 14 | + | 16319 | 16705 | 387 | 4 | >ref|ZP_05254816.1| two-component system response regulator [Bacteroides sp. 4_3_47FAA] | 2,0E-61 |
| 15 | + | 16889 | 17083 | 195 | 4 | >ref|ZP_05254816.1| two-component system response regulator [Bacteroides sp. 4_3_47FAA] | 3,0E-28 |
| 16 | + | 17101 | 17373 | 273 | 5 | >ref|ZP_05254816.1| two-component system response regulator [Bacteroides sp. 4_3_47FAA] | 2,0E-36 |
| 17 | + | 17406 | 17687 | 282 | 6 | >ref|ZP_05254816.1| two-component system response regulator [Bacteroides sp. 4_3_47FAA] | 4E-46 |
| 18 | + | 17720 | 19015 | 1296 | 6 | >ref|ZP_05254815.1| two-component system sensor histidine kinase [Bacteroides sp. 4_3_47FAA] | 0 |
| 19 | + | 19155 | 19391 | 237 | N/A | >ref|ZP_01959018.1| hypothetical protein BACCAC_00611 [Bacteroides caccae ATCC 43185] | 1,0E-07 |
| 20 | + | 19403 | 19918 | 516 | 7 | >ref|ZP_05254984.1| tyrosine type site-specific recombinase [Bacteroides sp. 4_3_47FAA] | 1,0E-23 |
| 21 | + | 19869 | 20645 | 777 | 7 | >ref|ZP_05761247.1| tyrosine type site-specific recombinase [Bacteroides sp. D2] | 3,0E-43 |
| 22 | - | 20791 | 20967 | 177 | 8 | >ref|ZP_05256799.1| conserved hypothetical protein [Bacteroides sp. 4_3_47FAA] | 2,0E-24 |
| 23 | - | 21317 | 22195 | 879 | 9 | >ref|YP_001299003.1| hypothetical protein BVU_1705 [Bacteroides vulgatus ATCC 8482] | 3,0E-140 |
| 24 | - | 22330 | 22554 | 225 | 10 | >ref|YP_001299004.1| hypothetical protein BVU_1706 [Bacteroides vulgatus ATCC 8482] | 1,0E-19 |
| 25 | + | 23769 | 24212 | 444 | 11 | >ref|ZP_01959463.1| hypothetical protein BACCAC_01067 [Bacteroides caccae ATCC 43185] | 8,0E-08 |
| 26 | - | 24356 | 24799 | 444 | 12 | >ref|ZP_03300252.1| hypothetical protein BACDOR_01619 [Bacteroides dorei DSM 17855] | 4,0E-74 |
| 27 | + | 24936 | 25799 | 864 | 13 | >ref|ZP_03642550.1| hypothetical protein BACCOPRO_00907 [Bacteroides coprophilus DSM 18228] | 1E-134 |
| 28 | + | 26070 | 26261 | 192 | 14 | >ref|ZP_02070312.1| hypothetical protein BACUNI_01732 [Bacteroides uniformis ATCC 8492] | 2,0E-04 |
| 29 | + | 26332 | 27453 | 1122 | 14 | >ref|ZP_03642551.1| hypothetical protein BACCOPRO_00908 [Bacteroides coprophilus DSM 18228] | 0 |
| 30 | - | 28014 | 28181 | 168 | 15 | >ref|ZP_05284340.1| hypothetical protein Bfra3_23942 [Bacteroides fragilis 3_1_12] | 4,0E-07 |
| 31 | - | 28381 | 28674 | 294 | 16 | >ref|ZP_05284340.1| hypothetical protein Bfra3_23942 [Bacteroides fragilis 3_1_12] | 3E-33 |
| 32 | + | 29435 | 30244 | 810 | 17 | >ref|ZP_06987183.1| dinitrogenase iron-molybdenum cofactor [Bacteroides sp. 3_1_19] | 3E-126 |
| 33 | - | 30318 | 30623 | 306 | 18 | >ref|ZP_06744076.1| conserved hypothetical protein [Bacteroides vulgatus PC510] | 2,0E-47 |
| 34 | + | 30829 | 32043 | 1215 | 19 | >ref|ZP_06744075.1| site-specific recombinase, phage integrase family [Bacteroides vulgatus PC510] | 0 |
| 35 | + | 32361 | 32648 | 288 | N/A | No significant similarity found | N/A |
| 36 | + | 32697 | 33314 | 618 | 20 | >ref|ZP_03009263.1| hypothetical protein BACCOP_01119 [Bacteroides coprocola DSM 17136] | 1,0E-64 |
| 37 | + | 33333 | 33857 | 525 | 20 | >ref|NP_904612.1| secretion activator protein, putative [Porphyromonas gingivalis W83] | 1,0E-58 |
| 38 | + | 33847 | 34380 | 534 | 20 | >ref|ZP_06744072.1| putative lipoprotein [Bacteroides vulgatus PC510] | 2,0E-77 |
| 39 | + | 34861 | 35058 | 198 | 21 | >ref|ZP_05257921.1| conserved hypothetical protein [Bacteroides sp. 4_3_47FAA] | 1,0E-22 |
| 40 | + | 35166 | 35321 | 156 | N/A | >ref|ZP_05257917.1| predicted protein [Bacteroides sp. 4_3_47FAA] | 6,0E-05 |
| 41 | + | 35327 | 35494 | 168 | 22 | No significant similarity found | N/A |
| 42 | + | 36124 | 36612 | 489 | 23 | >ref|ZP_06744077.1| hypothetical protein CUU_1439 [Bacteroides vulgatus PC510] | 2,0E-87 |
| 43 | + | 36658 | 36945 | 288 | 23 | >ref|ZP_06744078.1| hypothetical protein CUU_1440 [Bacteroides vulgatus PC510] | 3,0E-47 |
