# Supplementary material for: Functional Metagenomics: A High Throughput Screening Method to Decipher Microbiota-Driven NF-κB Modulation in the Human Gut
Source: PLoS One. 2010 Sep 30;5(9):e13092. doi: 10.1371/journal.pone.0013092 (PMC2948039; doi:10.1371/journal.pone.0013092)
Supplement: Table S3 — Phylogenetic indication of the first blastp result for predicted genes of 52B7 insert. Length is expressed in bp (base pairs). (0.06 MB DOC) [file pone.0013092.s005.doc]

Table S3. Phylogenetic indication of the first blastp result for predicted genes of 52B7 insert.

| **Gene** | **Length** | **Phylogenetic first blastp result** |
| --- | --- | --- |
| 1 | 1032 | [Bacteroides vulgatus ATCC 8482] |
| 2 | 888 | [Bacteroides vulgatus ATCC 8482] |
| 3 | 2352 | [Bacteroides sp. 4_3_47FAA] |
| 4 | 675 | [Bacteroides sp. 4_3_47FAA] |
| 5 | 486 | [Bacteroides vulgatus PC510] |
| 6 | 1917 | [Bacteroides vulgatus ATCC 8482] |
| 7 | 2370 | [Bacteroides vulgatus ATCC 8482] |
| 8 | 2397 | [Bacteroides vulgatus ATCC 8482] |
| 9 | 1251 | [Bacteroides vulgatus ATCC 8482] |
| 10 | 1470 | [Bacteroides sp. 4_3_47FAA] |
| 11 | 171 | [Bacteroides vulgatus ATCC 8482] |
| 12 | 273 | [Bacteroides vulgatus ATCC 8482] |
| 13 | 240 | [Bacteroides vulgatus ATCC 8482] |
| 14 | 387 | [Bacteroides vulgatus ATCC 8482] |
| 15 | 195 | [Bacteroides dorei DSM 17855] |
| 16 | 273 | [Bacteroides sp. 4_3_47FAA] |
| 17 | 282 | [Bacteroides vulgatus PC510] |
| 18 | 1296 | [Bacteroides vulgatus PC510] |
| 19 | 237 | [Bacteroides caccae ATCC 43185] |
| 20 | 516 | [Bacteroides vulgatus ATCC 8482] |
| 21 | 777 | [Bacteroides caccae ATCC 43185] |
| 22 | 177 | [Bacteroides sp. 4_3_47FAA] |
| 23 | 879 | [Bacteroides vulgatus ATCC 8482] |
| 24 | 225 | [Bacteroides vulgatus ATCC 8482] |
| 25 | 444 | [Bacteroides caccae ATCC 43185] |
| 26 | 444 | [Bacteroides dorei DSM 17855] |
| 27 | 864 | [Bacteroides coprophilus DSM 18228] |
| 28 | 192 | [Bacteroides sp. D20] |
| 29 | 1122 | [Bacteroides coprophilus DSM 18228] |
| 30 | 168 | [Bacteroides fragilis 3_1_12] |
| 31 | 294 | [Bacteroides fragilis 3_1_12] |
| 32 | 810 | [Parabacteroides distasonis ATCC 8503] |
| 33 | 306 | [Bacteroides vulgatus PC510] |
| 34 | 1215 | [Bacteroides vulgatus PC510] |
| 35 | 288 | Uncultured organism clone VC1CL59TR |
| 36 | 618 | [Bacteroides coprocola DSM 17136] |
| 37 | 525 | [Bacteroides sp. 9_1_42FAA] |
| 38 | 534 | [Bacteroides sp. 4_3_47FAA] |
| 39 | 198 | [Bacteroides sp. 4_3_47FAA] |
| 40 | 156 | [Bacteroides sp. 4_3_47FAA] |
| 41 | 168 | n/a |
| 42 | 489 | [Bacteroides sp. 4_3_47FAA] |
| 43 | 288 | [Bacteroides vulgatus PC510] |
