# Supplementary material for: Functional Metagenomics: A High Throughput Screening Method to Decipher Microbiota-Driven NF-κB Modulation in the Human Gut
Source: PLoS One. 2010 Sep 30;5(9):e13092. doi: 10.1371/journal.pone.0013092 (PMC2948039; doi:10.1371/journal.pone.0013092)
Supplement: Table S4 — The 52B7 metagenomic insert, key facts and figures. * bp: base pairs. (0.03 MB DOC) [file pone.0013092.s006.doc]

Table S4. The 52B7 metagenomic insert, key facts and figures.

|  | **length in bp*** | **% of total metagenomic DNA** | **% of coding metagenomic DNA** |
| --- | --- | --- | --- |
| 52B7 metagenomic DNA insert | 37006 | 100,00 | * |
| total gene length | 29889 | 80,77 | 100,00 |
| assigned to Bacteroides spp. | 29433 | 79,54 | 98,47 |
| assigned to Bacteroides vulgatus | 17391 | 47,00 | 58,19 |
| assigned to Bacteroides vulgatus ATCC 8482 | 12546 | 33,90 | 41,98 |
| assigned to Bacteroides sp. 4_3_47FAA | 6324 | 17,09 | 21,16 |
| assigned to Bacteroides vulgatus PC510 | 4845 | 13,09 | 16,21 |
