# Supplementary material for: Functional Metagenomics: A High Throughput Screening Method to Decipher Microbiota-Driven NF-κB Modulation in the Human Gut
Source: PLoS One. 2010 Sep 30;5(9):e13092. doi: 10.1371/journal.pone.0013092 (PMC2948039; doi:10.1371/journal.pone.0013092)
Supplement: Table S5 — TLRs expression in Caco-2/kb-seap-7. Results are expressed as MFI (Mean Fluorescence Intensity). (0.03 MB DOC) [file pone.0013092.s007.doc]

Table S5. TLRs expression in Caco-2/kb-seap-7.

| TLR | Membrane | Isotype Control (membrane) | Intracellular | Isotype Control  (intracellular) |
| --- | --- | --- | --- | --- |
| TLR2 | 4.30 | 3.97 |  |  |
| TLR3 | 6.35 | 4.54 | 11.08 | 3.81 |
| TLR4 | 9.13 | 3.97 |  |  |
| TLR5 | 23.99 | 3.97 |  |  |
| TLR6 | 18.5 | 4.54 |  |  |
| TLR7 |  |  | 8.71 | 4.77 |
| TLR8 |  |  | 14.11 | 3.81 |
| TLR9 | 5.9 | 4.9 | 10.48 | 4.20 |
