# Supplementary material for: Functional Metagenomics: A High Throughput Screening Method to Decipher Microbiota-Driven NF-κB Modulation in the Human Gut
Source: PLoS One. 2010 Sep 30;5(9):e13092. doi: 10.1371/journal.pone.0013092 (PMC2948039; doi:10.1371/journal.pone.0013092)
Supplement: Method S1 — Construction and use of Caco-2 reporter cells. (0.03 MB DOC) [file pone.0013092.s008.doc]

**Method S1. Construction and use of Caco-2 reporter cells.**

Caco-2 cells were transfected by electroporation with the pNiFty2-SEAP plasmid (Invivogen) using the Amaxa nucleofection system (Lonza). The Caco-2/kb-seap-7 clone was selected for its response to 10 ng/mL of IL-1β (Peprotech) after 24 h stimulation.

Caco-2 cells were cultured in DMEM (Sigma) supplemented with the same amounts of glutamine and antibiotics, 20 % heat-inactivated FCS and 1x non-essential amino acids (Invitrogen). For experiments, Caco-2/kb-seap-7 reporter cells were seeded at 50 000 cells per well, into 96-wells plates and incubated 24 hours before stimulation. Cells were stimulated with 10 µl of each tested substances with the final volume per well of 100 µl. Cells were stimulated with a concentration range of each inhibitor in the presence or absence of 10 ng/mL of IL-1β. SEAP in the supernatant was revealed using Quanti-Blue^TM^ reagent (Invivogen) using the manufacturer’s protocol and quantified as OD at 655nm. All measurements were performed using a microplate reader (Infinite 200, Tecan).
