# Supplementary material for: Functional Metagenomics: A High Throughput Screening Method to Decipher Microbiota-Driven NF-κB Modulation in the Human Gut
Source: PLoS One. 2010 Sep 30;5(9):e13092. doi: 10.1371/journal.pone.0013092 (PMC2948039; doi:10.1371/journal.pone.0013092)
Supplement: Method S2 — Measurment of IL-8 secretion. (0.02 MB DOC) [file pone.0013092.s009.doc]

**Method S2. Measurment of IL-8 secretion.**

Secretion of interleukine-8 (IL-8) was quantified by enzyme-linked immunosorbent assay (ELISA) using the human IL-8 ELISA development kit (Peprotech) according to manufacturer’s instructions. Experiments were performed on parental HT-29 cells and HT-29/kb-seap-25. Cells were seeded at 50 000 cells per well, into 96-wells plates and incubated 24 hours before stimulation. Cells were stimulated with 10 µl of each tested substances with the final volume per well of 100 µl and incubated 24 hours. Supernatants of stimulated HT-29 were collected and frozen until use. Bacterial supernatant of 52B7 was obtained after filtration (0.2 µm filter) of an overnight culture grown at 37°C in LB.
